# 2,11-Dibromo-5,8-dibut­yl[4]helicene

**DOI:** 10.1107/S1600536812013141

**Published:** 2012-03-31

**Authors:** Hiroyuki Isobe, Taisuke Matsuno, Shunpei Hitosugi, Waka Nakanishi

**Affiliations:** a Department of Chemistry, Tohoku University, Aoba-ku, Sendai 980-8578, Japan

## Abstract

A racemic mixture of the title compound, C_26_H_26_Br_2_, a brominated [4]helicene, crystallizes, forming columns of stacked mol­ecules. There are two crystallographically unique mol­ecules in the asymmetric unit, both with the same helical handedness. As is typical with helicene congeners, the unique mol­ecules show short inter­atomic contacts between H atoms at the fjord region, with H⋯H distances of 1.87 and 1.94 Å. Mol­ecules with the same helical handedness segregate in the crystal packing, forming homochiral columns. The stacked mol­ecules are piled in a column with alternate orientations. The shortest C⋯C distance in the stacked mol­ecules is 3.306 (4) Å.

## Related literature

For the synthesis, see: Ichikawa *et al.* (2008 ▶); Isobe *et al.* (2009 ▶); Nakanishi *et al.* (2011 ▶). For nonsubstituted [4]helicene, see: Hirshfeld *et al.* (1963 ▶). For halogenated [4]helicenes, see: Amsharov *et al.* (2009 ▶); Bae *et al.* (2007 ▶). For an optical application of stacking helicenes, see: Verbiest *et al.* (1998 ▶).
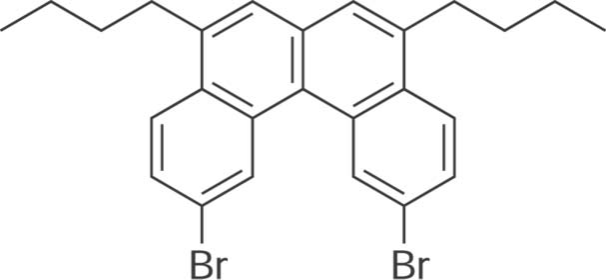


## Experimental

### 

#### Crystal data

C_26_H_26_Br_2_
*M* *_r_* = 498.29
Triclinic, 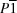

*a* = 7.611 (2) Å
*b* = 13.394 (4) Å
*c* = 22.552 (7) Å
α = 75.012 (4)°
β = 84.682 (4)°
γ = 79.067 (4)°
*V* = 2178.2 (11) Å^3^
*Z* = 4
Mo *K*α radiation
μ = 3.73 mm^−1^
*T* = 100 K
0.40 × 0.10 × 0.10 mm

#### Data collection

Bruker APEXII CCD area-detector diffractometer
Absorption correction: multi-scan (*SADABS*; Sheldrick, 1996 ▶) *T*_min_ = 0.317, *T*_max_ = 0.707
25036 measured reflections
10029 independent reflections
7642 reflections with *I* > 2σ(*I*)
*R*_int_ = 0.038

#### Refinement

*R*[*F* ^2^ > 2σ(*F* ^2^)] = 0.032
*wR*(*F* ^2^) = 0.069
*S* = 1.02
10029 reflections
509 parameters
H-atom parameters constrained
Δρ_max_ = 0.58 e Å^−3^
Δρ_min_ = −0.40 e Å^−3^

### 

Data collection: *APEX2* (Bruker, 2007 ▶); cell refinement: *SAINT* (Bruker, 2007 ▶); data reduction: *SAINT*; program(s) used to solve structure: *SHELXS97* (Sheldrick, 2008 ▶); program(s) used to refine structure: *SHELXL97* (Sheldrick, 2008 ▶); molecular graphics: *ORTEP-3* (Farrugia, 1997 ▶) and *Mercury* (Macrae *et al.*, 2008 ▶); software used to prepare material for publication: *SHELXL97*, Yadokari-XG 2009 (Kabuto *et al.*, 2009 ▶) and *publCIF* (Westrip, 2010 ▶).

## Supplementary Material

Crystal structure: contains datablock(s) I, global. DOI: 10.1107/S1600536812013141/nk2148sup1.cif

Structure factors: contains datablock(s) I. DOI: 10.1107/S1600536812013141/nk2148Isup2.hkl

Supplementary material file. DOI: 10.1107/S1600536812013141/nk2148Isup3.cml

Additional supplementary materials:  crystallographic information; 3D view; checkCIF report

## Figures and Tables

**Fig. 1.**
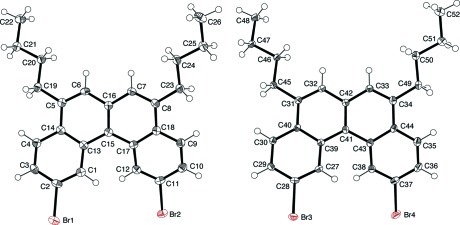
Molecular structures of the title compound with displacement ellipsoids drawn at the 50% probability level. Two non-equivalent molecules for (P)-form are shown. Note that the two structures differ only slightly at the methylene chain and that the enantiomeric (M)-form can be found as the mirror geometries.

**Fig. 2.**
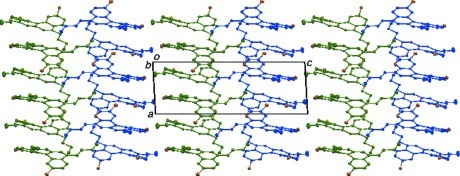
Packing structure of the title compound, viewed along the b axis. The carbon atoms of enantiomers are colored differently. Color code: C for (P)-form = green, C for (M)-form = blue, Br = brown. Hydrogen atoms are omitted for clarity. Note that one colomn comprise single enantiomeric molecules with an alternate stack of two crystallographically unique molecules.

**Fig. 3.**
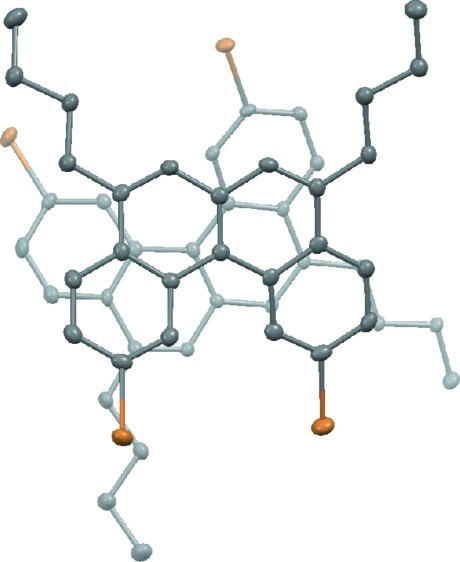
Stacking structure of the (P)-form. Hydrogen atoms are omitted for clarity.

## supplementary crystallographic information

### Comment

Columnar assembly of polycyclic aromatic hydrocarbons (PAH) is attractive in materials science. The assembly of helicenes, PAH with helically aligned *sp*^2^-carbons, is of particular interest for unique applications such as second-order nonlinear optical materials from [6]helicene (Verbiest *et al.*, 1998). Smaller helicenes, on the other hand, have been less attractive, as the molecule is too small for columnar assembly. For instance, the smallest helicene, [4]helicene, packs in a crystal with a herringbone motif (Hirshfeld *et al.*, 1963). Recently, a few examples of halogenated [4]helicenes have been reported (Amsharov *et al.*, 2009; Bae *et al.*, 2007; Isobe *et al.*, 2009), which indicate beneficial effects of halogen substituents for the stacking assembly. However, various forms of the stacking structures are found even among these rare examples. We found that additional effects from the substituents other than the halogen can affect the packing structure. We previously reported a crystallographic analysis of 2,11-dibromo-5,8-dimethyl[4]helicene and found that the enantiomers of 2,11-dibromo-5,8-dimethyl[4]helicene segregate to form homochiral columns of the stacking molecules (Isobe *et al.*, 2009). By changing the alkyl substituents at 5,8-position, we found a different form of columnar assembly. The asymmetric unit of the title compound is shown in Fig. 1. A set of alternate molecular pairs in a column is observed as non-equivalent molecules in the asymmetric unit. The interatomic distances between the hydrogen atoms at the fjord region are 1.87 Å and 1.94 Å. The packing structures are shown in Fig. 2 and 3. Each of the enantiomers segregate to form homochiral columns (Fig. 2). Unlike 5,8-dimethyl derivative that formed the stack with a synchronized orientation (Isobe *et al.*, 2009), the title compound with 5,8-dibutyl substituents formed the stack with an alternating orientation (Fig. 3) with the shortest intermolecular C···C distance of 3.306 (4) Å between C1 and C42 (Fig. 1).

### Experimental

The title compound was synthesized from difluoroalkene through acid-mediated intramolecular cyclization and dehydrogenative aromatization as reported in the literatures (Ichikawa *et al.*, 2008; Nakanishi *et al.*, 2011). A single-crystal suitable for X-ray crystallographic analysis was obtained by slow evaporation from a mixture of hexane and 2-propanol.

### Refinement

H atoms were included in calculated positions and treated as riding atoms, with C—H = 0.95, 0.99 and 0.98 Å for CH (aromatic), CH_2_ and CH_3_, respectively, with *U*_iso_(H) = 1.2*U*_eq_(C) for CH (aromatic) and CH_2_, and *U*_iso_(H) = 1.5*U*_eq_(C) for CH_3_.

### Crystal data

**Table:**

C_26_H_26_Br_2_	*Z* = 4
*M**_r_* = 498.29	*F*(000) = 1008
Triclinic, *P*1	*D*_x_ = 1.519 Mg m^−^^3^
Hall symbol: -P 1	Mo *K*α radiation, λ = 0.71073 Å
*a* = 7.611 (2) Å	Cell parameters from 7028 reflections
*b* = 13.394 (4) Å	θ = 2.7–27.8°
*c* = 22.552 (7) Å	µ = 3.73 mm^−^^1^
α = 75.012 (4)°	*T* = 100 K
β = 84.682 (4)°	Needle, colourless
γ = 79.067 (4)°	0.40 × 0.10 × 0.10 mm
*V* = 2178.2 (11) Å^3^	

### Data collection

**Table:**

Bruker APEXII CCD area-detector diffractometer	10029 independent reflections
Radiation source: Bruker TXS fine-focus rotating anode	7642 reflections with *I* > 2σ(*I*)
Bruker Helios multilayer confocal mirror monochromator	*R*_int_ = 0.038
Detector resolution: 8.333 pixels mm^-1^	θ_max_ = 28.0°, θ_min_ = 1.6°
phi and ω scans	*h* = −9→9
Absorption correction: multi-scan (*SADABS*; Sheldrick, 1996)	*k* = −17→17
*T*_min_ = 0.317, *T*_max_ = 0.707	*l* = −29→29
25036 measured reflections	

### Refinement

**Table:**

Refinement on *F*^2^	Primary atom site location: structure-invariant direct methods
Least-squares matrix: full	Secondary atom site location: difference Fourier map
*R*[*F*^2^ > 2σ(*F*^2^)] = 0.032	Hydrogen site location: inferred from neighbouring sites
*wR*(*F*^2^) = 0.069	H-atom parameters constrained
*S* = 1.02	*w* = 1/[σ^2^(*F*_o_^2^) + (0.0289*P*)^2^ + 0.3827*P*] where *P* = (*F*_o_^2^ + 2*F*_c_^2^)/3
10029 reflections	(Δ/σ)_max_ = 0.001
509 parameters	Δρ_max_ = 0.58 e Å^−^^3^
0 restraints	Δρ_min_ = −0.40 e Å^−^^3^

### Special details

**Table:**

Geometry. All e.s.d.'s (except the e.s.d. in the dihedral angle between two l.s. planes) are estimated using the full covariance matrix. The cell e.s.d.'s are taken into account individually in the estimation of e.s.d.'s in distances, angles and torsion angles; correlations between e.s.d.'s in cell parameters are only used when they are defined by crystal symmetry. An approximate (isotropic) treatment of cell e.s.d.'s is used for estimating e.s.d.'s involving l.s. planes.
Refinement. Refinement of *F*^2^ against ALL reflections. The weighted *R*-factor *wR* and goodness of fit *S* are based on *F*^2^, conventional *R*-factors *R* are based on *F*, with *F* set to zero for negative *F*^2^. The threshold expression of *F*^2^ > σ(*F*^2^) is used only for calculating *R*-factors(gt) *etc*. and is not relevant to the choice of reflections for refinement. *R*-factors based on *F*^2^ are statistically about twice as large as those based on *F*, and *R*- factors based on ALL data will be even larger.

### Fractional atomic coordinates and isotropic or equivalent isotropic displacement parameters (Å^2^)

**Table:**

	*x*	*y*	*z*	*U*_iso_*/*U*_eq_	
Br2	0.69711 (3)	0.70118 (2)	0.060451 (12)	0.02587 (7)	
Br1	1.16728 (4)	0.43519 (2)	0.260717 (13)	0.02937 (7)	
Br3	−0.05183 (3)	1.083930 (19)	0.395653 (12)	0.02066 (7)	
Br4	0.15219 (4)	1.263921 (19)	0.131483 (13)	0.02509 (7)	
C17	0.7427 (3)	0.84372 (19)	0.20139 (11)	0.0172 (5)	
C11	0.6991 (3)	0.8033 (2)	0.10549 (12)	0.0208 (6)	
C5	0.8009 (3)	0.76413 (19)	0.39852 (11)	0.0187 (5)	
C24	0.6615 (3)	1.22388 (19)	0.21135 (12)	0.0229 (6)	
H24	0.5651	1.2175	0.2441	0.027*	
H24A	0.7775	1.2054	0.2314	0.027*	
C6	0.7382 (3)	0.8623 (2)	0.36512 (11)	0.0196 (5)	
H6	0.6946	0.9155	0.3863	0.024*	
C9	0.6639 (3)	0.9822 (2)	0.10803 (11)	0.0216 (6)	
H9	0.6378	1.0547	0.0880	0.026*	
C14	0.8766 (3)	0.68580 (19)	0.36537 (12)	0.0178 (5)	
C2	1.0445 (3)	0.53591 (19)	0.30318 (12)	0.0206 (6)	
C20	0.7004 (3)	0.8207 (2)	0.49937 (11)	0.0215 (6)	
H20	0.7607	0.8828	0.4870	0.026*	
H20A	0.5765	0.8427	0.4852	0.026*	
C12	0.7336 (3)	0.7702 (2)	0.16657 (12)	0.0201 (5)	
H12	0.7516	0.6974	0.1860	0.024*	
C25	0.6396 (4)	1.3374 (2)	0.17339 (12)	0.0252 (6)	
H25	0.5173	1.3586	0.1578	0.030*	
H25A	0.7256	1.3418	0.1375	0.030*	
C23	0.6547 (3)	1.14628 (19)	0.17281 (11)	0.0204 (5)	
H23	0.5368	1.1645	0.1540	0.025*	
H23A	0.7472	1.1563	0.1389	0.025*	
C13	0.8691 (3)	0.70858 (19)	0.29984 (11)	0.0172 (5)	
C8	0.6825 (3)	1.03148 (19)	0.20605 (12)	0.0185 (5)	
C21	0.6942 (3)	0.7827 (2)	0.56915 (11)	0.0215 (6)	
H21	0.8181	0.7637	0.5834	0.026*	
H21A	0.6392	0.7186	0.5814	0.026*	
C19	0.7995 (3)	0.73642 (19)	0.46809 (11)	0.0200 (5)	
H19	0.9251	0.7185	0.4806	0.024*	
H19A	0.7448	0.6726	0.4838	0.024*	
C7	0.6867 (3)	0.99747 (19)	0.26827 (11)	0.0191 (5)	
H7	0.6565	1.0478	0.2921	0.023*	
C3	1.0465 (3)	0.5111 (2)	0.36730 (12)	0.0215 (6)	
H3	1.1022	0.4441	0.3897	0.026*	
C1	0.9613 (3)	0.63116 (19)	0.27019 (12)	0.0194 (5)	
H1	0.9654	0.6457	0.2266	0.023*	
C16	0.7346 (3)	0.88965 (19)	0.29932 (11)	0.0174 (5)	
C22	0.5882 (4)	0.8647 (2)	0.60081 (13)	0.0286 (6)	
H22	0.6393	0.9292	0.5878	0.043*	
H22A	0.5944	0.8378	0.6455	0.043*	
H22B	0.4629	0.8796	0.5895	0.043*	
C10	0.6659 (3)	0.9092 (2)	0.07497 (12)	0.0227 (6)	
H10	0.6450	0.9303	0.0323	0.027*	
C18	0.7001 (3)	0.95204 (19)	0.17166 (11)	0.0181 (5)	
C15	0.7811 (3)	0.81207 (19)	0.26662 (11)	0.0176 (5)	
C4	0.9660 (3)	0.58630 (19)	0.39673 (12)	0.0206 (5)	
H4	0.9702	0.5710	0.4402	0.025*	
C31	0.3858 (3)	0.68314 (18)	0.34132 (10)	0.0130 (5)	
C27	0.1242 (3)	0.95644 (18)	0.31852 (11)	0.0146 (5)	
H27	0.0674	1.0115	0.2869	0.018*	
C28	0.1018 (3)	0.96479 (18)	0.37815 (11)	0.0153 (5)	
C46	0.5611 (3)	0.49584 (18)	0.37603 (11)	0.0148 (5)	
H46	0.4770	0.4667	0.3570	0.018*	
H46A	0.6583	0.5145	0.3451	0.018*	
C34	0.2271 (3)	0.80180 (19)	0.12655 (11)	0.0155 (5)	
C43	0.2110 (3)	0.93667 (18)	0.18604 (11)	0.0151 (5)	
C47	0.6410 (3)	0.41243 (18)	0.43115 (11)	0.0164 (5)	
H47	0.7185	0.4430	0.4520	0.020*	
H47A	0.5432	0.3899	0.4607	0.020*	
C32	0.3822 (3)	0.66950 (18)	0.28404 (11)	0.0142 (5)	
H32	0.4288	0.6023	0.2770	0.017*	
C38	0.2047 (3)	1.04416 (18)	0.18422 (11)	0.0157 (5)	
H38	0.2313	1.0632	0.2197	0.019*	
C39	0.2304 (3)	0.86706 (18)	0.30324 (10)	0.0129 (5)	
C41	0.2525 (3)	0.85374 (18)	0.24114 (10)	0.0137 (5)	
C37	0.1605 (3)	1.12078 (18)	0.13162 (12)	0.0186 (5)	
C35	0.1400 (3)	0.99264 (19)	0.07767 (11)	0.0190 (5)	
H35	0.1184	0.9755	0.0410	0.023*	
C30	0.2797 (3)	0.79725 (19)	0.41311 (11)	0.0162 (5)	
H30	0.3333	0.7428	0.4456	0.019*	
C51	0.2167 (4)	0.6369 (2)	0.00702 (12)	0.0270 (6)	
H51	0.3125	0.6669	−0.0207	0.032*	
H51A	0.1001	0.6732	−0.0104	0.032*	
C33	0.2958 (3)	0.72843 (18)	0.17626 (10)	0.0148 (5)	
H33	0.3355	0.6584	0.1728	0.018*	
C44	0.1886 (3)	0.91036 (18)	0.13003 (10)	0.0147 (5)	
C42	0.3111 (3)	0.75217 (18)	0.23372 (11)	0.0136 (5)	
C49	0.1996 (3)	0.77430 (19)	0.06719 (11)	0.0187 (5)	
H49	0.2838	0.8064	0.0350	0.022*	
H49A	0.0766	0.8067	0.0543	0.022*	
C40	0.3019 (3)	0.78316 (18)	0.35275 (10)	0.0129 (5)	
C29	0.1832 (3)	0.88721 (19)	0.42630 (11)	0.0167 (5)	
H29	0.1723	0.8962	0.4670	0.020*	
C48	0.7509 (3)	0.31728 (19)	0.41193 (12)	0.0219 (6)	
H48	0.6729	0.2843	0.3935	0.033*	
H48A	0.8044	0.2667	0.4481	0.033*	
H48B	0.8460	0.3397	0.3818	0.033*	
C50	0.2256 (3)	0.65698 (19)	0.07008 (11)	0.0200 (5)	
H50	0.3433	0.6222	0.0871	0.024*	
H50A	0.1316	0.6257	0.0981	0.024*	
C36	0.1230 (3)	1.0964 (2)	0.07799 (12)	0.0212 (6)	
H36	0.0865	1.1503	0.0426	0.025*	
C45	0.4620 (3)	0.59480 (18)	0.39403 (10)	0.0144 (5)	
H45	0.3626	0.5757	0.4239	0.017*	
H45A	0.5454	0.6209	0.4152	0.017*	
C26	0.6699 (4)	1.4139 (2)	0.20959 (14)	0.0331 (7)	
H26	0.5832	1.4114	0.2447	0.050*	
H26A	0.6544	1.4852	0.1829	0.050*	
H26B	0.7917	1.3943	0.2245	0.050*	
C52	0.2384 (4)	0.5200 (2)	0.00990 (14)	0.0354 (7)	
H52	0.3515	0.4834	0.0286	0.053*	
H52A	0.2392	0.5104	−0.0318	0.053*	
H52B	0.1384	0.4912	0.0347	0.053*	

### Atomic displacement parameters (Å^2^)

**Table:**

	*U*^11^	*U*^22^	*U*^33^	*U*^12^	*U*^13^	*U*^23^
Br2	0.02828 (15)	0.02909 (16)	0.02485 (15)	−0.00958 (11)	0.00169 (11)	−0.01247 (12)
Br1	0.02749 (15)	0.02402 (15)	0.03654 (17)	0.00126 (11)	0.00085 (12)	−0.01227 (13)
Br3	0.02107 (13)	0.01674 (13)	0.02431 (15)	0.00260 (10)	−0.00162 (10)	−0.00935 (11)
Br4	0.03039 (15)	0.01316 (13)	0.02919 (16)	−0.00265 (10)	−0.00684 (11)	0.00038 (11)
C17	0.0124 (12)	0.0211 (14)	0.0191 (13)	−0.0049 (10)	0.0028 (9)	−0.0066 (11)
C11	0.0158 (12)	0.0281 (15)	0.0231 (15)	−0.0081 (11)	0.0034 (10)	−0.0130 (12)
C5	0.0159 (12)	0.0212 (14)	0.0199 (14)	−0.0077 (10)	−0.0008 (10)	−0.0033 (11)
C24	0.0257 (14)	0.0182 (14)	0.0229 (15)	−0.0035 (11)	−0.0013 (11)	−0.0019 (12)
C6	0.0182 (13)	0.0223 (14)	0.0222 (14)	−0.0062 (10)	0.0017 (10)	−0.0113 (12)
C9	0.0199 (13)	0.0235 (14)	0.0197 (14)	−0.0020 (11)	0.0009 (10)	−0.0040 (12)
C14	0.0111 (11)	0.0186 (13)	0.0249 (14)	−0.0070 (10)	−0.0006 (10)	−0.0047 (11)
C2	0.0136 (12)	0.0188 (14)	0.0318 (16)	−0.0044 (10)	0.0036 (10)	−0.0108 (12)
C20	0.0215 (13)	0.0213 (14)	0.0223 (14)	−0.0048 (11)	−0.0028 (10)	−0.0052 (12)
C12	0.0154 (12)	0.0192 (14)	0.0247 (15)	−0.0036 (10)	0.0015 (10)	−0.0043 (11)
C25	0.0271 (14)	0.0209 (14)	0.0258 (15)	−0.0034 (11)	−0.0028 (11)	−0.0024 (12)
C23	0.0192 (13)	0.0198 (14)	0.0216 (14)	−0.0022 (10)	−0.0028 (10)	−0.0040 (11)
C13	0.0128 (12)	0.0173 (13)	0.0225 (14)	−0.0079 (10)	0.0010 (10)	−0.0036 (11)
C8	0.0122 (12)	0.0176 (13)	0.0247 (14)	−0.0015 (10)	−0.0012 (10)	−0.0043 (11)
C21	0.0200 (13)	0.0234 (14)	0.0214 (14)	−0.0064 (11)	−0.0004 (10)	−0.0039 (12)
C19	0.0206 (13)	0.0203 (14)	0.0186 (14)	−0.0073 (10)	0.0004 (10)	−0.0018 (11)
C7	0.0179 (13)	0.0179 (13)	0.0224 (14)	−0.0017 (10)	−0.0014 (10)	−0.0074 (11)
C3	0.0180 (13)	0.0153 (13)	0.0294 (16)	−0.0043 (10)	−0.0022 (11)	−0.0010 (12)
C1	0.0143 (12)	0.0229 (14)	0.0226 (14)	−0.0077 (10)	0.0022 (10)	−0.0063 (11)
C16	0.0127 (12)	0.0202 (13)	0.0207 (14)	−0.0050 (10)	−0.0010 (10)	−0.0056 (11)
C22	0.0265 (15)	0.0306 (16)	0.0308 (16)	−0.0051 (12)	0.0029 (12)	−0.0125 (13)
C10	0.0188 (13)	0.0294 (15)	0.0195 (14)	−0.0045 (11)	0.0011 (10)	−0.0058 (12)
C18	0.0129 (12)	0.0217 (14)	0.0190 (14)	−0.0048 (10)	0.0003 (10)	−0.0028 (11)
C15	0.0131 (12)	0.0197 (13)	0.0209 (14)	−0.0058 (10)	0.0007 (10)	−0.0048 (11)
C4	0.0208 (13)	0.0199 (14)	0.0213 (14)	−0.0066 (10)	−0.0007 (10)	−0.0035 (11)
C31	0.0113 (11)	0.0134 (12)	0.0135 (12)	−0.0030 (9)	0.0008 (9)	−0.0019 (10)
C27	0.0138 (12)	0.0112 (12)	0.0179 (13)	−0.0010 (9)	−0.0023 (9)	−0.0021 (10)
C28	0.0148 (12)	0.0109 (12)	0.0211 (13)	−0.0011 (9)	0.0013 (9)	−0.0069 (10)
C46	0.0157 (12)	0.0132 (12)	0.0140 (13)	−0.0005 (9)	−0.0012 (9)	−0.0017 (10)
C34	0.0126 (11)	0.0200 (13)	0.0134 (13)	−0.0030 (9)	0.0007 (9)	−0.0034 (10)
C43	0.0117 (11)	0.0171 (13)	0.0147 (13)	−0.0014 (9)	−0.0015 (9)	−0.0015 (10)
C47	0.0185 (12)	0.0129 (12)	0.0156 (13)	−0.0004 (9)	−0.0005 (10)	−0.0017 (10)
C32	0.0140 (11)	0.0114 (12)	0.0159 (13)	−0.0013 (9)	0.0002 (9)	−0.0019 (10)
C38	0.0142 (12)	0.0178 (13)	0.0148 (13)	−0.0025 (9)	−0.0011 (9)	−0.0037 (10)
C39	0.0114 (11)	0.0130 (12)	0.0145 (12)	−0.0037 (9)	−0.0001 (9)	−0.0029 (10)
C41	0.0115 (11)	0.0173 (13)	0.0120 (12)	−0.0032 (9)	−0.0007 (9)	−0.0024 (10)
C37	0.0184 (12)	0.0119 (12)	0.0234 (14)	−0.0024 (10)	−0.0007 (10)	−0.0007 (11)
C35	0.0176 (12)	0.0218 (14)	0.0161 (13)	−0.0017 (10)	−0.0027 (10)	−0.0027 (11)
C30	0.0189 (12)	0.0147 (13)	0.0143 (13)	−0.0021 (10)	−0.0037 (9)	−0.0019 (10)
C51	0.0380 (16)	0.0217 (15)	0.0223 (15)	−0.0033 (12)	−0.0067 (12)	−0.0068 (12)
C33	0.0154 (12)	0.0137 (12)	0.0145 (13)	−0.0023 (9)	0.0012 (9)	−0.0031 (10)
C44	0.0141 (12)	0.0153 (12)	0.0121 (12)	−0.0028 (9)	0.0000 (9)	0.0010 (10)
C42	0.0123 (11)	0.0147 (12)	0.0138 (12)	−0.0034 (9)	0.0003 (9)	−0.0033 (10)
C49	0.0222 (13)	0.0197 (14)	0.0133 (13)	−0.0022 (10)	−0.0035 (10)	−0.0026 (11)
C40	0.0115 (11)	0.0128 (12)	0.0134 (12)	−0.0027 (9)	−0.0018 (9)	−0.0008 (10)
C29	0.0211 (13)	0.0173 (13)	0.0132 (13)	−0.0044 (10)	−0.0003 (10)	−0.0054 (10)
C48	0.0239 (14)	0.0164 (13)	0.0225 (14)	0.0013 (10)	−0.0012 (11)	−0.0029 (11)
C50	0.0235 (13)	0.0207 (14)	0.0153 (13)	−0.0038 (11)	−0.0024 (10)	−0.0031 (11)
C36	0.0221 (13)	0.0185 (14)	0.0178 (14)	−0.0004 (10)	−0.0034 (10)	0.0033 (11)
C45	0.0145 (12)	0.0156 (13)	0.0133 (12)	−0.0012 (9)	−0.0015 (9)	−0.0047 (10)
C26	0.0366 (17)	0.0202 (15)	0.0436 (19)	−0.0071 (12)	0.0009 (14)	−0.0089 (14)
C52	0.055 (2)	0.0232 (16)	0.0306 (17)	−0.0064 (14)	−0.0089 (14)	−0.0102 (14)

### Geometric parameters (Å, º)

**Table:**

Br2—C11	1.906 (2)	C31—C40	1.446 (3)
Br1—C2	1.906 (2)	C31—C45	1.513 (3)
Br3—C28	1.898 (2)	C27—C28	1.371 (3)
Br4—C37	1.905 (2)	C27—C39	1.414 (3)
C17—C18	1.421 (3)	C27—H27	0.9500
C17—C12	1.423 (3)	C28—C29	1.393 (3)
C17—C15	1.463 (3)	C46—C47	1.525 (3)
C11—C12	1.367 (4)	C46—C45	1.528 (3)
C11—C10	1.393 (4)	C46—H46	0.9900
C5—C6	1.356 (3)	C46—H46A	0.9900
C5—C14	1.445 (3)	C34—C33	1.357 (3)
C5—C19	1.515 (3)	C34—C44	1.449 (3)
C24—C25	1.526 (3)	C34—C49	1.519 (3)
C24—C23	1.528 (3)	C43—C38	1.422 (3)
C24—H24	0.9900	C43—C44	1.429 (3)
C24—H24A	0.9900	C43—C41	1.450 (3)
C6—C16	1.435 (3)	C47—C48	1.525 (3)
C6—H6	0.9500	C47—H47	0.9900
C9—C10	1.372 (3)	C47—H47A	0.9900
C9—C18	1.424 (3)	C32—C42	1.430 (3)
C9—H9	0.9500	C32—H32	0.9500
C14—C4	1.413 (3)	C38—C37	1.374 (3)
C14—C13	1.434 (3)	C38—H38	0.9500
C2—C1	1.368 (3)	C39—C40	1.427 (3)
C2—C3	1.398 (4)	C39—C41	1.448 (3)
C20—C21	1.523 (3)	C41—C42	1.397 (3)
C20—C19	1.527 (3)	C37—C36	1.398 (3)
C20—H20	0.9900	C35—C36	1.372 (3)
C20—H20A	0.9900	C35—C44	1.414 (3)
C12—H12	0.9500	C35—H35	0.9500
C25—C26	1.526 (4)	C30—C29	1.375 (3)
C25—H25	0.9900	C30—C40	1.412 (3)
C25—H25A	0.9900	C30—H30	0.9500
C23—C8	1.510 (3)	C51—C50	1.523 (3)
C23—H23	0.9900	C51—C52	1.528 (4)
C23—H23A	0.9900	C51—H51	0.9900
C13—C1	1.418 (3)	C51—H51A	0.9900
C13—C15	1.461 (3)	C33—C42	1.431 (3)
C8—C7	1.360 (3)	C33—H33	0.9500
C8—C18	1.451 (3)	C49—C50	1.531 (3)
C21—C22	1.526 (3)	C49—H49	0.9900
C21—H21	0.9900	C49—H49A	0.9900
C21—H21A	0.9900	C29—H29	0.9500
C19—H19	0.9900	C48—H48	0.9800
C19—H19A	0.9900	C48—H48A	0.9800
C7—C16	1.426 (3)	C48—H48B	0.9800
C7—H7	0.9500	C50—H50	0.9900
C3—C4	1.366 (3)	C50—H50A	0.9900
C3—H3	0.9500	C36—H36	0.9500
C1—H1	0.9500	C45—H45	0.9900
C16—C15	1.399 (3)	C45—H45A	0.9900
C22—H22	0.9800	C26—H26	0.9800
C22—H22A	0.9800	C26—H26A	0.9800
C22—H22B	0.9800	C26—H26B	0.9800
C10—H10	0.9500	C52—H52	0.9800
C4—H4	0.9500	C52—H52A	0.9800
C31—C32	1.354 (3)	C52—H52B	0.9800
			
C18—C17—C12	117.5 (2)	C27—C28—C29	122.1 (2)
C18—C17—C15	119.6 (2)	C27—C28—Br3	118.65 (18)
C12—C17—C15	122.8 (2)	C29—C28—Br3	119.19 (18)
C12—C11—C10	122.2 (2)	C47—C46—C45	112.13 (19)
C12—C11—Br2	119.0 (2)	C47—C46—H46	109.2
C10—C11—Br2	118.81 (19)	C45—C46—H46	109.2
C6—C5—C14	117.7 (2)	C47—C46—H46A	109.2
C6—C5—C19	122.2 (2)	C45—C46—H46A	109.2
C14—C5—C19	120.1 (2)	H46—C46—H46A	107.9
C25—C24—C23	112.8 (2)	C33—C34—C44	117.9 (2)
C25—C24—H24	109.0	C33—C34—C49	122.2 (2)
C23—C24—H24	109.0	C44—C34—C49	119.8 (2)
C25—C24—H24A	109.0	C38—C43—C44	117.9 (2)
C23—C24—H24A	109.0	C38—C43—C41	122.3 (2)
H24—C24—H24A	107.8	C44—C43—C41	119.6 (2)
C5—C6—C16	123.1 (2)	C48—C47—C46	111.6 (2)
C5—C6—H6	118.4	C48—C47—H47	109.3
C16—C6—H6	118.4	C46—C47—H47	109.3
C10—C9—C18	121.6 (2)	C48—C47—H47A	109.3
C10—C9—H9	119.2	C46—C47—H47A	109.3
C18—C9—H9	119.2	H47—C47—H47A	108.0
C4—C14—C13	118.6 (2)	C31—C32—C42	122.6 (2)
C4—C14—C5	120.5 (2)	C31—C32—H32	118.7
C13—C14—C5	120.9 (2)	C42—C32—H32	118.7
C1—C2—C3	121.9 (2)	C37—C38—C43	120.6 (2)
C1—C2—Br1	119.31 (19)	C37—C38—H38	119.7
C3—C2—Br1	118.80 (19)	C43—C38—H38	119.7
C21—C20—C19	112.4 (2)	C27—C39—C40	117.3 (2)
C21—C20—H20	109.1	C27—C39—C41	122.7 (2)
C19—C20—H20	109.1	C40—C39—C41	119.7 (2)
C21—C20—H20A	109.1	C42—C41—C39	117.5 (2)
C19—C20—H20A	109.1	C42—C41—C43	117.2 (2)
H20—C20—H20A	107.9	C39—C41—C43	125.3 (2)
C11—C12—C17	120.8 (2)	C38—C37—C36	121.7 (2)
C11—C12—H12	119.6	C38—C37—Br4	119.17 (19)
C17—C12—H12	119.6	C36—C37—Br4	119.14 (18)
C24—C25—C26	113.2 (2)	C36—C35—C44	122.3 (2)
C24—C25—H25	108.9	C36—C35—H35	118.8
C26—C25—H25	108.9	C44—C35—H35	118.8
C24—C25—H25A	108.9	C29—C30—C40	122.1 (2)
C26—C25—H25A	108.9	C29—C30—H30	118.9
H25—C25—H25A	107.7	C40—C30—H30	118.9
C8—C23—C24	116.6 (2)	C50—C51—C52	112.1 (2)
C8—C23—H23	108.1	C50—C51—H51	109.2
C24—C23—H23	108.1	C52—C51—H51	109.2
C8—C23—H23A	108.1	C50—C51—H51A	109.2
C24—C23—H23A	108.1	C52—C51—H51A	109.2
H23—C23—H23A	107.3	H51—C51—H51A	107.9
C1—C13—C14	117.4 (2)	C34—C33—C42	122.7 (2)
C1—C13—C15	123.3 (2)	C34—C33—H33	118.7
C14—C13—C15	119.2 (2)	C42—C33—H33	118.7
C7—C8—C18	117.0 (2)	C35—C44—C43	118.5 (2)
C7—C8—C23	122.9 (2)	C35—C44—C34	121.2 (2)
C18—C8—C23	120.1 (2)	C43—C44—C34	120.1 (2)
C20—C21—C22	112.8 (2)	C41—C42—C32	120.4 (2)
C20—C21—H21	109.0	C41—C42—C33	120.6 (2)
C22—C21—H21	109.0	C32—C42—C33	118.9 (2)
C20—C21—H21A	109.0	C34—C49—C50	115.8 (2)
C22—C21—H21A	109.0	C34—C49—H49	108.3
H21—C21—H21A	107.8	C50—C49—H49	108.3
C5—C19—C20	116.3 (2)	C34—C49—H49A	108.3
C5—C19—H19	108.2	C50—C49—H49A	108.3
C20—C19—H19	108.2	H49—C49—H49A	107.4
C5—C19—H19A	108.2	C30—C40—C39	119.1 (2)
C20—C19—H19A	108.2	C30—C40—C31	120.8 (2)
H19—C19—H19A	107.4	C39—C40—C31	120.0 (2)
C8—C7—C16	122.9 (2)	C30—C29—C28	117.9 (2)
C8—C7—H7	118.5	C30—C29—H29	121.0
C16—C7—H7	118.5	C28—C29—H29	121.0
C4—C3—C2	118.0 (2)	C47—C48—H48	109.5
C4—C3—H3	121.0	C47—C48—H48A	109.5
C2—C3—H3	121.0	H48—C48—H48A	109.5
C2—C1—C13	121.2 (2)	C47—C48—H48B	109.5
C2—C1—H1	119.4	H48—C48—H48B	109.5
C13—C1—H1	119.4	H48A—C48—H48B	109.5
C15—C16—C7	121.1 (2)	C51—C50—C49	112.0 (2)
C15—C16—C6	120.4 (2)	C51—C50—H50	109.2
C7—C16—C6	118.5 (2)	C49—C50—H50	109.2
C21—C22—H22	109.5	C51—C50—H50A	109.2
C21—C22—H22A	109.5	C49—C50—H50A	109.2
H22—C22—H22A	109.5	H50—C50—H50A	107.9
C21—C22—H22B	109.5	C35—C36—C37	118.4 (2)
H22—C22—H22B	109.5	C35—C36—H36	120.8
H22A—C22—H22B	109.5	C37—C36—H36	120.8
C9—C10—C11	118.4 (2)	C31—C45—C46	115.26 (19)
C9—C10—H10	120.8	C31—C45—H45	108.5
C11—C10—H10	120.8	C46—C45—H45	108.5
C17—C18—C9	119.2 (2)	C31—C45—H45A	108.5
C17—C18—C8	120.8 (2)	C46—C45—H45A	108.5
C9—C18—C8	119.8 (2)	H45—C45—H45A	107.5
C16—C15—C13	117.4 (2)	C25—C26—H26	109.5
C16—C15—C17	116.5 (2)	C25—C26—H26A	109.5
C13—C15—C17	126.0 (2)	H26—C26—H26A	109.5
C3—C4—C14	122.9 (2)	C25—C26—H26B	109.5
C3—C4—H4	118.6	H26—C26—H26B	109.5
C14—C4—H4	118.6	H26A—C26—H26B	109.5
C32—C31—C40	118.3 (2)	C51—C52—H52	109.5
C32—C31—C45	121.9 (2)	C51—C52—H52A	109.5
C40—C31—C45	119.6 (2)	H52—C52—H52A	109.5
C28—C27—C39	121.0 (2)	C51—C52—H52B	109.5
C28—C27—H27	119.5	H52—C52—H52B	109.5
C39—C27—H27	119.5	H52A—C52—H52B	109.5
			
C14—C5—C6—C16	3.6 (3)	C39—C27—C28—C29	0.8 (3)
C19—C5—C6—C16	−178.6 (2)	C39—C27—C28—Br3	−177.06 (17)
C6—C5—C14—C4	171.4 (2)	C45—C46—C47—C48	−175.9 (2)
C19—C5—C14—C4	−6.5 (3)	C40—C31—C32—C42	4.8 (3)
C6—C5—C14—C13	−6.1 (3)	C45—C31—C32—C42	−179.9 (2)
C19—C5—C14—C13	176.1 (2)	C44—C43—C38—C37	6.4 (3)
C10—C11—C12—C17	−2.1 (4)	C41—C43—C38—C37	−178.4 (2)
Br2—C11—C12—C17	177.71 (17)	C28—C27—C39—C40	4.5 (3)
C18—C17—C12—C11	5.1 (3)	C28—C27—C39—C41	177.9 (2)
C15—C17—C12—C11	−179.3 (2)	C27—C39—C41—C42	−160.5 (2)
C23—C24—C25—C26	−172.6 (2)	C40—C39—C41—C42	12.7 (3)
C25—C24—C23—C8	177.7 (2)	C27—C39—C41—C43	16.6 (3)
C4—C14—C13—C1	−3.5 (3)	C40—C39—C41—C43	−170.2 (2)
C5—C14—C13—C1	174.1 (2)	C38—C43—C41—C42	−160.0 (2)
C4—C14—C13—C15	−178.4 (2)	C44—C43—C41—C42	15.2 (3)
C5—C14—C13—C15	−0.9 (3)	C38—C43—C41—C39	22.9 (3)
C24—C23—C8—C7	9.6 (3)	C44—C43—C41—C39	−161.9 (2)
C24—C23—C8—C18	−172.4 (2)	C43—C38—C37—C36	−1.0 (4)
C19—C20—C21—C22	−177.4 (2)	C43—C38—C37—Br4	179.58 (17)
C6—C5—C19—C20	8.1 (3)	C44—C34—C33—C42	7.7 (3)
C14—C5—C19—C20	−174.1 (2)	C49—C34—C33—C42	−175.7 (2)
C21—C20—C19—C5	175.6 (2)	C36—C35—C44—C43	3.5 (3)
C18—C8—C7—C16	10.0 (3)	C36—C35—C44—C34	−172.3 (2)
C23—C8—C7—C16	−172.1 (2)	C38—C43—C44—C35	−7.5 (3)
C1—C2—C3—C4	−1.6 (4)	C41—C43—C44—C35	177.1 (2)
Br1—C2—C3—C4	176.61 (18)	C38—C43—C44—C34	168.3 (2)
C3—C2—C1—C13	−1.6 (4)	C41—C43—C44—C34	−7.0 (3)
Br1—C2—C1—C13	−179.74 (17)	C33—C34—C44—C35	171.3 (2)
C14—C13—C1—C2	4.1 (3)	C49—C34—C44—C35	−5.4 (3)
C15—C13—C1—C2	178.8 (2)	C33—C34—C44—C43	−4.4 (3)
C8—C7—C16—C15	0.3 (4)	C49—C34—C44—C43	178.9 (2)
C8—C7—C16—C6	178.8 (2)	C39—C41—C42—C32	−13.0 (3)
C5—C6—C16—C15	6.2 (4)	C43—C41—C42—C32	169.7 (2)
C5—C6—C16—C7	−172.4 (2)	C39—C41—C42—C33	165.1 (2)
C18—C9—C10—C11	1.9 (4)	C43—C41—C42—C33	−12.2 (3)
C12—C11—C10—C9	−1.5 (4)	C31—C32—C42—C41	4.4 (3)
Br2—C11—C10—C9	178.69 (18)	C31—C32—C42—C33	−173.7 (2)
C12—C17—C18—C9	−4.7 (3)	C34—C33—C42—C41	0.8 (3)
C15—C17—C18—C9	179.6 (2)	C34—C33—C42—C32	178.9 (2)
C12—C17—C18—C8	171.6 (2)	C33—C34—C49—C50	9.4 (3)
C15—C17—C18—C8	−4.1 (3)	C44—C34—C49—C50	−174.0 (2)
C10—C9—C18—C17	1.3 (4)	C29—C30—C40—C39	3.6 (3)
C10—C9—C18—C8	−175.0 (2)	C29—C30—C40—C31	−172.6 (2)
C7—C8—C18—C17	−7.8 (3)	C27—C39—C40—C30	−6.5 (3)
C23—C8—C18—C17	174.1 (2)	C41—C39—C40—C30	179.9 (2)
C7—C8—C18—C9	168.4 (2)	C27—C39—C40—C31	169.6 (2)
C23—C8—C18—C9	−9.6 (3)	C41—C39—C40—C31	−3.9 (3)
C7—C16—C15—C13	165.5 (2)	C32—C31—C40—C30	171.3 (2)
C6—C16—C15—C13	−13.0 (3)	C45—C31—C40—C30	−4.2 (3)
C7—C16—C15—C17	−12.3 (3)	C32—C31—C40—C39	−4.8 (3)
C6—C16—C15—C17	169.2 (2)	C45—C31—C40—C39	179.71 (19)
C1—C13—C15—C16	−164.3 (2)	C40—C30—C29—C28	1.7 (3)
C14—C13—C15—C16	10.3 (3)	C27—C28—C29—C30	−4.0 (3)
C1—C13—C15—C17	13.3 (4)	Br3—C28—C29—C30	173.86 (17)
C14—C13—C15—C17	−172.1 (2)	C52—C51—C50—C49	−178.7 (2)
C18—C17—C15—C16	13.9 (3)	C34—C49—C50—C51	−173.3 (2)
C12—C17—C15—C16	−161.5 (2)	C44—C35—C36—C37	2.0 (4)
C18—C17—C15—C13	−163.7 (2)	C38—C37—C36—C35	−3.3 (4)
C12—C17—C15—C13	20.9 (4)	Br4—C37—C36—C35	176.13 (18)
C2—C3—C4—C14	2.1 (4)	C32—C31—C45—C46	9.6 (3)
C13—C14—C4—C3	0.4 (3)	C40—C31—C45—C46	−175.10 (19)
C5—C14—C4—C3	−177.1 (2)	C47—C46—C45—C31	177.42 (19)

## References

[bb1] Amsharov, K. Y., Kabdulov, M. A. & Jansen, M. (2009). *Eur. J. Org. Chem.* pp. 6328–6335.

[bb2] Bae, S., Mah, H., Chaturvedi, S., Jeknic, T. M., Baird, W. M., Katz, A. K., Carrell, H. L., Glusker, J. P., Okazaki, T., Laali, K. K., Zajc, B. & Lakshman, M. K. (2007). *J. Org. Chem.* **72**, 7625–7633. 10.1021/jo071145s 17764198

[bb3] Bruker (2007). *APEX2* and *SAINT* Bruker AXS Inc., Madison, Wisconsin, USA.

[bb4] Farrugia, L. J. (1997). *J. Appl. Cryst.* **30**, 565.

[bb5] Hirshfeld, F. L., Sandler, S. & Schmidt, G. M. J. (1963). *J. Chem. Soc.* 2108–2125.

[bb6] Ichikawa, J., Yokota, M., Kudo, T. & Umezaki, S. (2008). *Angew. Chem. Int. Ed.* **47**, 4870–4873. 10.1002/anie.200801396 18506861

[bb7] Isobe, H., Hitosugi, S., Matsuno, T., Iwamoto, T. & Ichikawa, J. (2009). *Org. Lett.* **11**, 4026–4028. 10.1021/ol901693y 19663471

[bb8] Kabuto, C., Akine, S., Nemoto, T. & Kwon, E. (2009). *J. Cryst. Soc. Jpn*, **51**, 218–224.

[bb9] Macrae, C. F., Bruno, I. J., Chisholm, J. A., Edgington, P. R., McCabe, P., Pidcock, E., Rodriguez-Monge, L., Taylor, R., van de Streek, J. & Wood, P. A. (2008). *J. Appl. Cryst.* **41**, 466–470.

[bb10] Nakanishi, W., Matsuno, T., Ichikawa, J. & Isobe, H. (2011). *Angew. Chem. Int. Ed.* **50**, 6048–6051. 10.1002/anie.201102210 21604350

[bb11] Sheldrick, G. M. (1996). *SADABS* University of Göttingen, Germany.

[bb12] Sheldrick, G. M. (2008). *Acta Cryst.* A**64**, 112–122. 10.1107/S0108767307043930 18156677

[bb13] Verbiest, T., Elshocht, S. V., Kauranen, M., Hellemans, L., Snauwaert, J., Nuckolls, C., Katz, T. J. & Persoons, A. (1998). *Science*, **30**, 913–915. 10.1126/science.282.5390.913 9794754

[bb14] Westrip, S. P. (2010). *J. Appl. Cryst.* **43**, 920–925.

